# Chromosome-Level Genome Assembly of *Morchella sextelata* Reveals Its Early Divergence and Adaptive Evolution

**DOI:** 10.3390/jof12050352

**Published:** 2026-05-10

**Authors:** Linhai Hong, Qi Fan, Nan Tao, Peng Wang, Ping Liu, Jing Leng, Chunxin Yao, Qinghong Liu

**Affiliations:** 1 Department of Vegetables, College of Horticulture, China Agricultural University, Beijing 100193, China; hlh@cau.edu.cn (L.H.); wangpeng_1998@cau.edu.cn (P.W.); 2 Biotechnology and Germplasm Resources Institute, Yunnan Academy of Agricultural Sciences, Kunming 650205, China; fanqi_666@163.com (Q.F.); tn1953@126.com (N.T.); liuping0606@126.com (P.L.); lengjing_2020@126.com (J.L.)

**Keywords:** *Morchella sextelata*, chromosome-level genome, assembly, adaptive evolution

## Abstract

This study presents a high-quality chromosome-level genome assembly of *Morchella sextelata* (54.64 Mb, 26 pseudochromosomes) and systematically characterizes its genomic and evolutionary features. Phylogenetic analysis indicates that *M. sextelata* diverged early within the Morchella genus (~14.2 million years ago) and underwent substantial genomic remodeling, with 1124 expanded and 1961 contracted gene families. Enrichment analysis of rapidly expanded gene families highlights two prominent functional themes: genes associated with small molecule/ion binding and secondary metabolite biosynthesis, and genes linked to the Fanconi anemia pathway and DNA repair/recombination. Notably, 56.96% of the COG-annotated *M. sextelata*-specific genes encode retrotransposon-related proteins, and this enrichment coincides with the expansion of DNA repair systems—a pattern reminiscent of the “transposon domestication” model. Functional genomic analyses further reveal that the glycoside hydrolase system is dominated by GH5, GH43, and GH3 families, suggesting a predicted capacity for plant cell wall polysaccharide degradation, while 12 biosynthetic gene clusters indicate genetic potential for terpenoid and non-ribosomal peptide biosynthesis. These findings provide a valuable genomic resource for *M. sextelata* and offer new insights into the role of transposable element mediated remodeling in fungal genome evolution.

## 1. Introduction

True morels (*Morchella* spp.), belonging to the phylum Ascomycota, are highly valued for their distinctive crisp texture, savory flavor, and rich nutritional profile [1]. They contain diverse bioactive compounds including carbohydrates, proteins, free amino acids, fatty acids, minerals, polyphenols, and alkaloids [2,3]. Extensive researches have demonstrated that morels exhibit various pharmacological properties, such as anti-fatigue, anti-inflammatory, and anti-tumor activities [3,4,5,6,7,8,9]. By 2022, the cultivation area for morels in China had expanded to approximately 16,466 hectares [10]. The primary commercially cultivated species include *M. sextelata*, *M. eximia*, and *M. importuna*, with *M. sextelata* dominating the Chinese market, accounting for over 85% of the national cultivation area [11]. Despite these advances, artificial cultivation of morels continues to face significant challenges, particularly regarding the elucidation of their growth regulation mechanisms. Omics technologies hold significant potential for unraveling the genetic blueprint of *Morchella*. A thorough genomic understanding is essential, as it will not only reveal the molecular mechanisms governing growth but also provide a scientific foundation for driving the sustainable development of the morel industry.

The genomic information of various *Morchella* species has been progressively reported in recent years. In 2018, the genomes of two *M. importuna* strains M04M24 and M04M26 were sequenced, with sizes of 48.98 Mb and 51.07 Mb, respectively [12]. Subsequently, the genome of *M. sextelata* was characterized, comprising 59 contigs totaling 52.93 Mb [13]. In 2020, a subchromosome-scale genome of *M. crassipes* was released, consisting of 23 scaffolds and spanning 56.76 Mb [14]. More recently, the whole-genome sequence of *M. eohespera* strain m200 was reported, with a size of 53.81 Mb [15]. Additionally, genomic data from wild morel species have been documented, such as a genome belonging to the Esculenta clade was published in 2024, which consists of 55.17 Mb [16]. Although these reports represent important progress, the current genomic resources for *Morchella* remain limited in several key aspects. Significant gaps persist in assembly continuity, completeness, and annotation depth. For instance, the assemblies of *M. sextelata*, *M. eohespera*, and *M. importuna* remain at the contig level, while only *M. crassipes* has reached scaffold-level resolution. Notably, no chromosome-level genome assembly has been achieved for *M. sextelata* to date, which hinders detailed comparative genomics and functional genomic investigations.

As the most widely cultivated *Morchella* species in China, *M. sextelata* is of paramount importance to the industry. However, the genetic basis underlying its key traits remains largely unexplored. Critically, the gene clusters and regulatory networks controlling fruiting body development, flavor compound synthesis, and environmental stress responses are systematically unresolved [17,18,19,20,21,22]. Furthermore, the unique biosynthetic potential of its secondary metabolites awaits in-depth exploration, a task fundamentally dependent on high-quality genomic resources [23,24,25]. Therefore, generating a high-quality, chromosome-level genome and conducting a systematic evolutionary analysis for *M. sextelata* are pivotal to elucidating its genetic basis, bridging the current knowledge gaps, and providing actionable insights for the sustainable advancement of the morel industry.

This study selected *M. sextelata* as the target species and successfully constructed the first chromosome-level reference genome for this strain by integrating PacBio HiFi sequencing, Hi-C chromosome conformation capture technology, and Illumina sequencing. The workflow included high-quality genome assembly, scaffolding, and comprehensive annotation. Systematic comparative genomic analyses with closely related species were performed to reveal genomic structural characteristics and evolutionary events. The high-quality genome of *M. sextelata* generated in this study not only serves as a valuable genetic resource but also provides a critical framework for future research. It deepens the understanding of genome evolution, physiological metabolism, and developmental biology within the *Morchella* genus.

## 2. Materials and Methods

### 2.1. Preparation of Sample

The *M. sextelata* strain m-ty1 used in this study was obtained from the Yunnan Academy of Agricultural Sciences and acquired via single-spore cultivation. Mycelial pellets were prepared by culturing the strain in PDB medium under dark conditions at 25 °C with shaking for 5 days. High-quality genomic DNA was extracted using the QIAGEN Genomic-tip kit, followed by quality assessment and accurate quantification with Nanodrop and Qubit. After passing quality control, the DNA was used for subsequent sequencing experiments. Total RNA was extracted following the protocol as previously described [26].

### 2.2. Genome Sequencing and Assembly

Illumina NovaSeq 6000 platform was used for short-read sequencing. For whole-genome DNA sequencing, libraries were prepared following the guidelines outlined in sample preparation guide (#15026486, Illumina, San Diego, CA, USA) and sequenced on this platform, yielding 21,969,257 reads (150 bp; ~120× coverage). The Hi-C library was constructed using the Hi-C Library Preparation Kit (Grandomics, Beijing, China) following the manufacturer’s instructions. Library quality was assessed prior to sequencing [27]. Hi-C sequencing produced 51,276,928 raw paired-end reads (150 bp; ~280× coverage). RNA-seq libraries were prepared using the TruSeq RNA Library Preparation Kit (Illumina, San Diego, CA, USA) according to the manufacturer’s protocol and sequenced on the same platform, yielding 29,330,374 reads (150 bp; ~160× coverage). Third-generation sequencing was performed on the PacBio Revio system using the SMRTbell Prep Kit 3.0 (Pacific Biosciences, Menlo Park, CA, USA) for DNA library preparation which generated 143,739 HiFi reads (average length 22,509 bp; ~60× coverage).

Data quality control was performed using Fastp (v0.23.2) [28] to filter Illumina sequencing data by removing low-quality reads and adapter sequences. Specifically, a sliding window-based quality filtering was performed with a minimum base quality score of 20. Reads containing any ambiguous base ‘N’ were discarded. Additionally, a fixed-length trimming strategy was applied: 5 nucleotides were cropped from both the 5′ end and the 3′ end of each read to remove potentially biased bases. Genome size was then estimated via K-mer-based analysis using Jellyfish (v2.2.10) and GenomeScope (v2.0.1) [29,30].

Genome assembly was performed using a hybrid approach. PacBio reads were assembled using hifiasm (v0.20.0) with Hi-C data support [31] and, independently, using Flye (v2.9.5) without Hi-C data [32]. Both assemblies were polished using Racon (v1.4.20) and Illumina short-read data [33,34]. Hifiasm employs a string-graph-based algorithm that excels in resolving heterozygous regions and generating highly contiguous primary contigs. Flye uses a repeat-aware overlap-layout-consensus (OLC) approach and is particularly effective for assembling repetitive genomic regions. To leverage the complementary strengths of both assemblers, the resulting assemblies were merged using Quickmerge (v0.3) [35], retaining the hifiasm primary contigs as the backbone and incorporating additional sequences from Flye to fill gaps and recover regions absent in the hifiasm assembly. Hi-C data were aligned to the merged assembly using Bwa (v0.7.18) [36]. Contigs clustering, ordering, and orientation were carried out with Juicer (v1.6), 3D-DNA (v201008), and Juicebox (v1.11.08) [37,38,39]. Redundant contigs were removed based on interaction patterns, and potential haplotigs were identified and removed by inspecting Hi-C contact maps for regions with abnormally high coverage and ambiguous interaction patterns in Juicebox. Gaps were filled with Ns, and telomere positions were identified using tidk [40]. Genome completeness was evaluated against the fungi_odb10 and ascomycota_odb10 datasets using BUSCO (v5.7.1) [41].

In the final assembly, the 38 contigs were organized into 33 scaffolds after Hi-C scaffolding, with five gaps remaining. The 33 scaffolds were subsequently arranged into 26 pseudochromosomes (LG1–LG26) based on Hi-C contact frequency patterns. The contig N50 and scaffold N50 are identical (1.94 Mb) because the five gaps are distributed across shorter scaffolds that do not affect the N50 statistic; the longest scaffolds each consist of a single contig. Hi-C scaffolding was validated by visual inspection of the genome-wide contact map, which showed a strong diagonal signal with minimal inter-chromosomal contacts, confirming accurate clustering and orientation (Figure S1).

### 2.3. Genome Annotation

To identify repetitive sequences in the *M. sextelata* genome, a de novo repetitive element database was first constructed using RepeatModeler (v2.0.5) [42], followed by the removal of redundant LTR sequences with LTR_retriever (v3.0.1) [43]. Based on this de novo repetitive element database and the Repbase database (version 20181026), repeats were annotated using RepeatMasker (v4.1.7) [44]. All subsequent analyses were performed on the resulting soft-masked genome.

Gene structures were predicted by integrating RNA-seq and homology-based evidence. RNA-seq reads were aligned to the genome using HISAT2 (v2.2.1) [45], and de novo transcripts were assembled with Trinity (v2.15.2) [46]. For homology evidence, protein sequences from nine related species were retrieved from the NCBI database (National Center for Biotechnology Information), including *M*. *conica* (GCA_003790465.1), *M. importuna* (GCF_003444635.1), *M. sextelata* (GCA_020137385.1), *M. snyderi* (GCA_024521645.1), *Aspergillus nidulans* (GCF_000011425.1), *Neurospora crassa* (GCF_000182925.2), *Peziza echinospora* (GCA_024516245.1), *Pyronema domesticum* (GCA_024516145.1), and *Saccharomyces cerevisiae* (GCA_000146045.2). These files were integrated by Funannotate (v1.8.17) [47] using the predict and update commands. The de novo gene structure prediction within this pipeline was performed by Augustus (v3.5.0) [48]. The completeness of the predicted protein sequences was evaluated against the fungi_odb10 and ascomycota_odb10 datasets using BUSCO (v5.7.1). Telomeric repeat sequences were predicted using tidk (v0.2.63) [40] with default parameters. The most abundant telomeric repeat motif was AACCCTAACT.

Functional annotation of the genome was performed using the funannotate annotate command. This integrated analysis included the prediction of Pfam domains, CAZymes, secreted proteins, proteases (MEROPS), BUSCO taxonomic profiles, and results from external tools: InterProScan (v5.76-107.0) [49], EggNOG-mapper (v2.1.13) [50], Phobius [51], SignalP (v6.0) [52], and antiSMASH (v8.0) [53].

### 2.4. Comparative Genomic Analysis

Orthologous gene families were identified across 12 fungal species using OrthoFinder (v2.5.4) [54]. The genomes and their accession numbers were as follows: *M. sextelata* (this study, strain m-ty1), *M. eximia* (GCA_024713935.1), *M. importuna* (GCF_003444635.1), *M. snyderi* (GCA_024521645.1), *M. conica* (GCA_003790465.1), *A*. *nidulans* (GCF_000011425.1), *A*. *niger* (GCA_000002855.2), *N*. *crassa* (GCF_000182925.2), *P*. *echinospora* (GCA_024516245.1), *P*. *domesticum* (GCA_024516145.1), *Pleurotus ostreatus* (GCA_014466165.1), and *S*. *cerevisiae* (GCA_000146045.2). Single-copy orthologs identified by OrthoFinder (excluding those from *P. ostreatus* and *S. cerevisiae*) were used for phylogenetic reconstruction. Their protein sequences were aligned using MUSCLE v5.2 [55] and concatenated into a supermatrix. A maximum likelihood phylogeny was then inferred with RAxML v8.2.13 [56] under the PROTGAMMAWAG model.

The MCMCtree (v4.9) from the PAML package was employed to estimate the divergence times between species [57]. Two time calibration nodes were the divergence time between *A*. *niger* and *N*. *crassa* (233.8–367.0 MYA) and the divergence time between Pezizomycotina and *N*. *crassa* (387.7–723.0 MYA) obtained from the TimeTree database [58]. A strict molecular clock model (clock = 1) was employed, with the LG + Γ substitution model (α = 0.5, four rate categories). Branch-specific substitution rates and the Hessian matrix were first estimated using codeml, and MCMCtree was then run with approximate likelihood calculation (usedata = 3). The MCMC chain was run for 20,100,000 generations, with the first 100,000 generations discarded as burn-in and sampling every 1000 generations, yielding 20,000 posterior samples. Convergence was assessed using Tracer (v1.7), ensuring effective sample sizes (ESS) > 200 for all estimated parameters.

Gene family evolution was assessed using CAFE v4.2.1 [59] with a species tree (branch length in Myr) and a count matrix of 14,866 families. A single birth-death rate (λ) was estimated under a uniform model. Families with FDR-corrected *p* < 0.05 were considered significantly expanded or contracted. Rapidly evolving gene families were defined as those with branch-specific *p* < 0.05 (FDR-corrected) and at least one rate shift on the *M. sextelata* branch identified by the Viterbi algorithm; these were further classified as rapidly expanding (net gain) or rapidly contracting (net loss).

To evaluate intraspecific nucleotide diversity among *M. sextelata* strains, the chromosome-level assembly generated in this study (m-ty1) was compared with three previously published *M. sextelata* assemblies (Mei et al., 2019 [13]; GCA_020137385.1; GCA_024713665.1) using ska (v1.0) [60]. Split k-mer files were constructed with “ska fasta”, and pairwise average nucleotide identity (ANI) and single nucleotide polymorphism (SNP) counts were computed with “ska distance”. Nucleotide divergence was calculated as the number of SNPs divided by the total number of aligned base pairs.

## 3. Results

### 3.1. Genome Assembly and Structural Annotation

**Figure 1:**
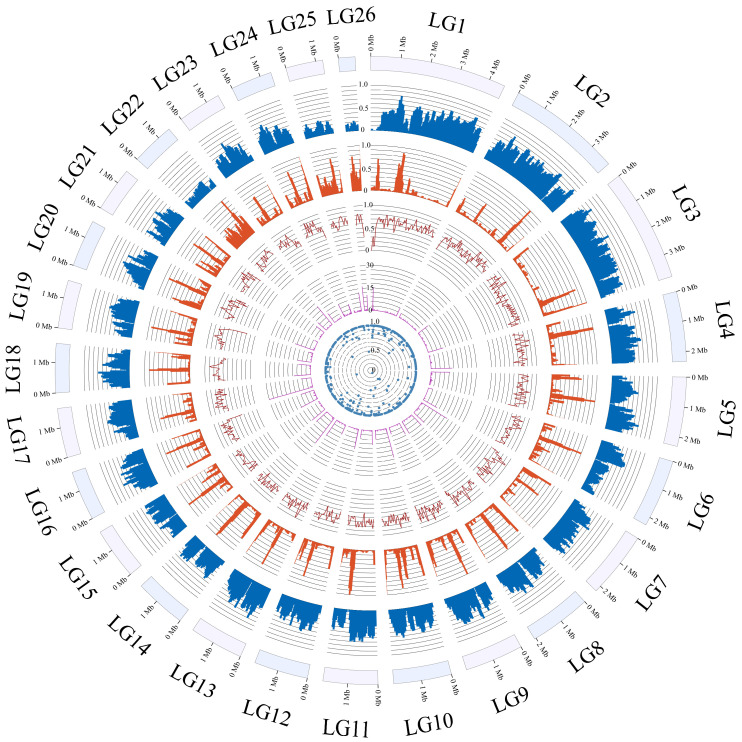
Distribution of the basic genomic elements of *M. sextelata*. The circles (outer to inner) represent: pseudochromosomes (LG1 to LG26); gene density, repetitive sequences density, GC content within the genome, distribution of telomere repeats and rate of sequencing coverage. Gene density, repetitive sequence density, GC content, and sequencing coverage depth are displayed as normalized values on a 0–1 scale (value/maximum value per track). Telomere repeats are shown as counts per window. All tracks were calculated using 100 kb sliding windows with a 50 kb step size.

In this study, we achieved a chromosome-level genome assembly of *M. sextelata* by integrating Illumina, PacBio Revio sequencing technologies, and Hi-C scaffolding. To attain a high-quality assembly, we merged the assemblies generated by both Flye and Hifiasm software, followed by optimization using Illumina data. The final assembly comprises 38 contigs assembled into 33 scaffolds, with a total length of 54.64 Mb and a scaffold N50 of 1.94 Mb (Table 1). Utilizing Hi-C data, the genome was anchored onto 26 pseudochromosomes (Figure 1 and Figure S1). Telomeric repeat sequences (AACCCTAACT) were identified at both ends of 16 pseudochromosomes, while the remaining 10 exhibited telomeres at only one end. Through the integration of de novo prediction, homology-based alignment, and RNA-seq evidence, a total of 11,269 protein-coding genes were predicted, with an average gene length (including introns and UTRs) of 1916.98 bp. BUSCO assessment (based on the fungi_odb10 database) of genome completeness and predicted protein set integrity yielded scores of 98.8% and 96.3%, respectively (Table 1).

Repetitive sequence annotation of the *M. sextelata* genome was conducted using a combination of de novo prediction and homology-based alignment. The total repetitive sequence content was 10.37 Mb, representing 18.96% of the genome. Notably, long terminal repeats (LTRs) comprised 8.36% of the entire genome and accounted for 44.09% of all repetitive elements. Further analysis of LTRs included estimating their insertion times based on sequence divergence within the LTR regions. By establishing a linear relationship between insertion frequency and time, we observed that their age distribution followed a characteristic “L-shaped” pattern (Figure S2).

### 3.2. Genome Comparison with Previously Published M. sextelata Assemblies

To assess genomic divergence among *M. sextelata* strains, we compared our chromosome-level assembly (m-ty1) with three previously published assemblies using ska. The ANI values between m-ty1 and the 2019, 2021, and 2022 assemblies were 99.83%, 99.86%, and 99.76%, respectively, indicating high nucleotide-level conservation across strains (Table 2). A total of 29,368, 20,604, and 28,212 SNPs were identified in the respective pairwise comparisons. Despite this overall conservation, the observed SNP densities (0.45–0.65 SNPs per kb) indicate a moderate degree of intraspecific nucleotide polymorphism among *M. sextelata* strains.

### 3.3. Phylogenetic and Gene Family Evolution Analysis

**Figure 2:**
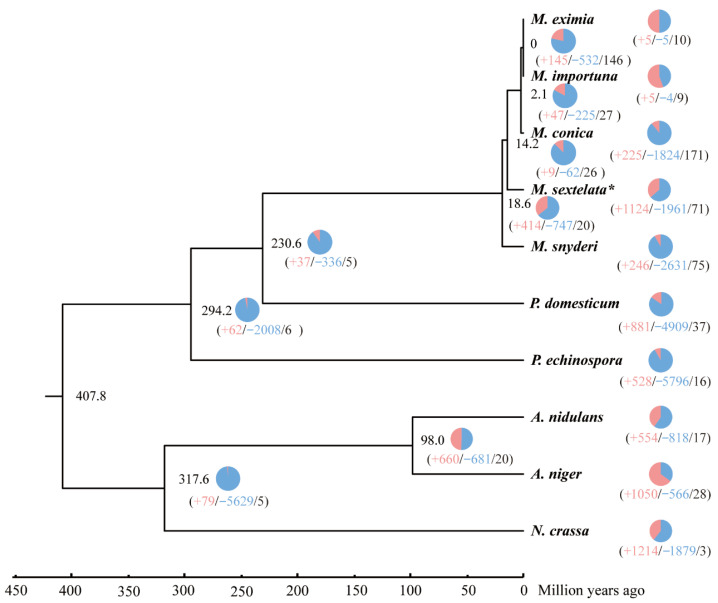
Phylogenetic relationship and divergence time for the newly assembled *M. sextelata* genome in this study and other fungus species. The number outside the parentheses represents the divergence time, while the three numbers inside the parentheses represent the number of expanded gene families, contracted gene families, and rapidly evolving gene families, respectively. * represents the species used in this study.

Phylogenetic tree reconstruction based on single-copy gene families from 10 fungal species (Figure 2) demonstrated that five *Morchella* species formed a distinct clade. Their divergence time was estimated to range from 0 to 18.6 million years ago (MYA), with *M. sextelata* diverging approximately 14.2 MYA. The analysis revealed that *M. snyderi* was the first to diverge from this lineage, followed by an early divergence of *M. sextelata*, indicating that *M. sextelata* represents one of the earlier-diverging species within the genus *Morchella*.

Gene family evolution analysis further revealed dynamic genomic changes in *M. sextelata* (Figure 2). Compared to its ancestral node, *M. sextelata* exhibited 1124 expanded gene families and 1961 contracted gene families, with contraction events predominating. Additionally, 71 rapidly evolving gene families were identified, including 50 rapidly expanding and 21 rapidly contracting families. Among the five analyzed *Morchella* species, *M. sextelata* showed the highest number of gene family expansions, while its contraction count was intermediate—greater than three species but lower than *M. snyderi*. Notably, across the *Morchella* evolutionary clade, most species displayed a general trend of gene family contractions exceeding expansions. These findings suggest that during evolution, *M. sextelata* underwent significant genomic remodeling, characterized by both massive losses of ancestral genes and the acquisition of new genes.

**Figure 3:**
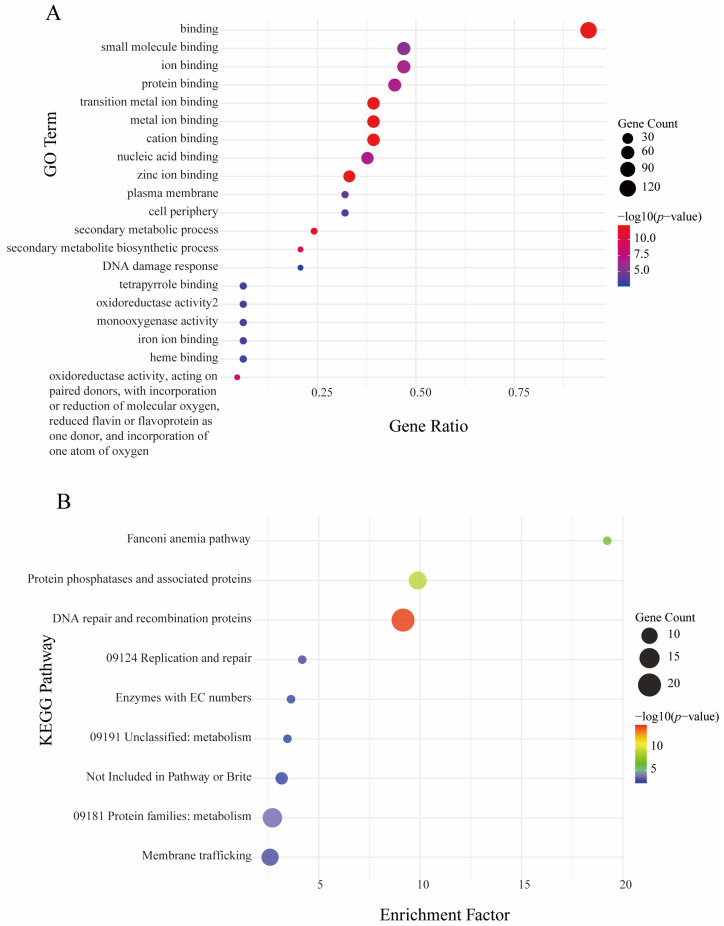
Rapidly expanding gene GO enrichment and KEGG pathway enrichment bubble plot. (**A**) GO enrichment analysis, showing the top 15 significantly enriched GO terms. The *x*-axis shows the Gene Ratio (k/n, where k = genes in the term, n = total input genes). Bubble size reflects the number of enriched genes, and color represents the −log_10_ (*p*-value); (**B**) KEGG pathway enrichment. The *x*-axis shows the Enrichment Factor (observed proportion divided by expected proportion; >1 indicates enrichment). Bubble size indicates gene count; color indicates −log_10_ (*p*-value).

To further investigate the functions of rapidly expanded gene families in *M. sextelata*, we performed Gene Ontology (GO) enrichment and Kyoto Encyclopedia of Genes and Genomes (KEGG) pathway analyses on the corresponding genes. GO enrichment analysis (Figure 3A) revealed significant enrichment in binding-related processes, particularly within molecular function categories including small molecule binding, ion binding, protein binding, and transition metal ion binding. Additionally, the biological process level exhibited notable enrichment for secondary metabolite biosynthetic processes. KEGG pathway analysis (Figure 3B) further uncovered functional specialization of these expanded gene families at the pathway level. Significantly enriched pathways included the Fanconi anemia pathway, DNA repair and recombination proteins, and protein phosphatases with associated proteins. Integrating these functional annotations, the rapidly expanded gene families in *M. sextelata* demonstrate two prominent functional themes: genes involved in small molecule/ion binding and secondary metabolite biosynthesis, and genes associated with DNA repair, recombination pathways and cell cycle regulation.

### 3.4. Unique Gene Families in M. sextelata

To investigate the evolutionary characteristics and gene family composition of *M. sextelata*, we conducted a comparative genomic analysis with 11 phylogenetically diverse fungal species. A total of 14,866 gene families were annotated across all species, encompassing 116,735 genes. Among these, 2669 gene families were conserved across all examined species, including 1379 single-copy orthologous groups. Within the *M. sextelata* genome, 9138 gene families were identified, of which 58 were unique lineages containing 200 genes (Table S2).

**Figure 4:**
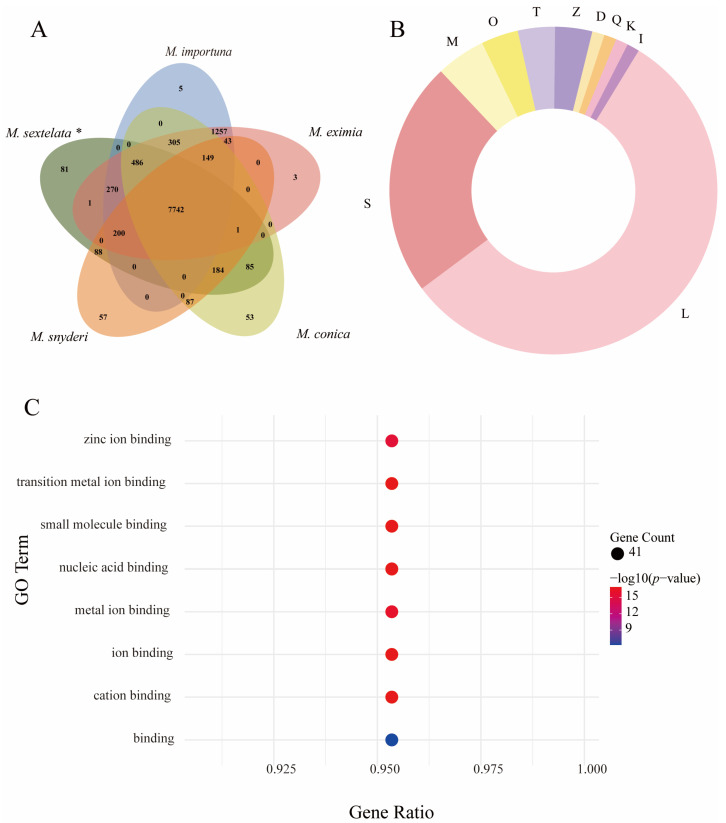
Analysis of unique gene families in *M. sextelata*. (**A**) Venn diagram comparing gene families across five *Morchella* species. * represents the species used in this study. (**B**) COG classification of unique genes in *M. sextelata*. Categories shown include: (L) Replication, recombination and repair; (S) Function unknown; (M) Cell wall/membrane/envelope biogenesis; (O) Post-translational modification, protein turnover, chaperones; (T) Signal transduction mechanisms; (Z) Cytoskeleton; (D) Cell cycle control, cell division, chromosome partitioning; (Q) Secondary metabolites biosynthesis, transport and catabolism; (K) Transcription; (I) Lipid transport and metabolism. (**C**) GO enrichment analysis of unique genes in *M. sextelata*. The *x*-axis shows the Gene Ratio (k/n, where k = genes in the term, n = total input genes). Bubble size reflects the number of enriched genes, and color represents the −log_10_ (*p*-value).

To further elucidate phylogenetic relationships within *Morchella*, the genome of *M. sextelata* was comparatively analyzed with four congeneric species (*M. eximia*, *M. conica*, *M. snyderi*, and *M. importuna*) (Figure 4A). This analysis identified 7742 core gene families conserved across all five *Morchella* species. Beyond this shared complement, *M. sextelata* possessed 486 gene families overlapping with three *Morchella* species (excluding *M. snyderi*). Notably, *M. sextelata* exhibited 81 unique gene families comprising 252 genes (Table S4).

Functional annotation via the Clusters of Orthologous Groups (COG) database (Figure 4B) [61] assigned 79 genes to functional categories, with 45 genes (58.2%) specifically associated with replication, recombination and repair (COG category L). The majority of these genes belonged to a single, rapidly expanding gene family (Table S2). GO enrichment analysis (Figure 4C) revealed significant enrichment in molecular binding activities, including zinc ion binding, transition metal ion binding, small molecule binding, nucleic acid binding, and metal ion binding.

Domain architecture analysis demonstrated that genes annotated to replication/recombination functions predominantly encode two conserved domains (Table S2): Retrotransposon gag protein (PF03732; 42 genes) and Ty3 transposon capsid-like protein (PF19259; 22 genes).

### 3.5. Functional Annotation of Genes and Compositional Features of CAZymes

In this study, systematic functional annotation was performed on the *M. sextelata* genome. Classification via the COG database (Figure S3) indicated that 2238 genes remained functionally uncharacterized. The predominant functional category among annotated genes was post-translational modification, protein turnover, and chaperones (686 genes; COG category O), critical for regulating protein activity and function [61]. Additionally, 496 genes were associated with carbohydrate transport and metabolism (COG category G), of which 377 genes encoded 388 carbohydrate-active enzymes (CAZymes) (Table S6).

**Figure 5:**
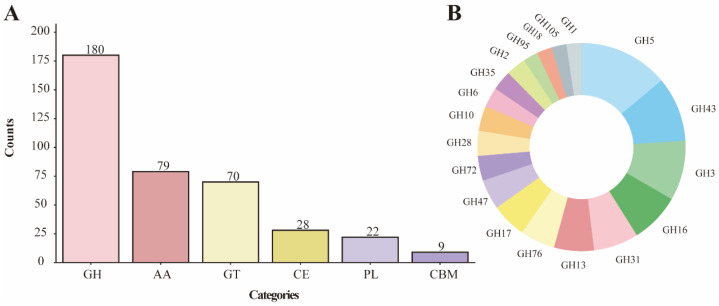
CAZymes composition characteristics of *M. sextelata*. (**A**) Number of main categories of CAZymes. (**B**) Proportion of each glycoside hydrolase (GH) family (with a count greater than 3).

CAZymes play central roles in fungal carbon source utilization and cell wall metabolism [62]. Genome-encoded CAZymes were classified into six major categories (Figure 5A): glycoside hydrolases (GHs; 180 genes, 47.5%) were predominant, followed by auxiliary activities (AAs; 79 genes) and glycosyltransferases (GTs; 70 genes). Among GH families, GH5 (endo-β-1,4-glucanase), GH43 (β-xylosidase), and GH3 (β-glucosidase) showed high abundance (Figure 5B). The presence of carbohydrate esterases (CEs; 28 genes) and polysaccharide lyases (PLs; 22 genes) suggests potential for degradation of complex polysaccharides such as pectin. Notably, AA families included multiple laccases (AA1) and aryl alcohol oxidases (AA3), which are predicted to function in lignin modification/degradation or oxidative stress responses based on sequence homology (Table S3).

### 3.6. Analysis of Secondary Metabolite Gene Clusters

Through antiSMASH analysis, a total of 152 genes implicated in secondary metabolite biosynthesis were identified, among which 90 genes were organized into 12 biosynthetic gene clusters (BGCs), comprising: 4 terpene BGCs, 2 terpene-precursor BGCs, 4 NRPS-like BGCs, 1 NRP-metallophore/NRPS BGC, and 1 hybrid NRPS-like/T1PKS BGC (Table 3, Table S5).

Notably, the Region 11 cluster exhibited 50% sequence similarity to the known clavaric acid biosynthetic cluster (an anti-tumor agent), while remaining clusters showed no significant homology to characterized pathways. This demonstrates *M. sextelata*’s genetic capacity for producing diverse secondary metabolites, with pronounced specialization in terpenoid and nonribosomal peptide biosynthesis.

## 4. Discussion

### 4.1. Obtaining a Chromosome-Level Genome of M. sextelata and Comparison with Previous Assemblies

The genome assembly presented in this study represents a substantial advancement over previously published *M. sextelata* genomes. Key improvements include: (i) Chromosome-level organization—this is the first *M. sextelata* genome anchored onto 26 pseudochromosomes, achieved through Hi-C scaffolding, whereas prior assemblies remained at the contig level [13] (GCA_020137385.1, GCA_024713665.1). (ii) Improved contiguity—the contig N50 (1.94 Mb) exceeds that of previous assemblies, with fewer scaffolds. (iii) Enhanced gene annotation—we predicted 11,269 protein-coding genes, substantially more than the 9550 genes in the first *M. sextelata* assembly [13], supported by integration of RNA-seq and homology evidence.

To evaluate intraspecific nucleotide diversity among *M. sextelata* strains, we compared our assembly (m-ty1) with the three previously published assemblies. Whole-genome alignment using ska identified average nucleotide identities (ANI) of 99.83%, 99.86%, and 99.76% relative to the 2019, 2021, and 2022 assemblies, respectively, indicating high genome-wide conservation. A total of 29,368, 20,604, and 28,212 SNPs were identified in the respective comparisons. These values correspond to SNP densities of approximately 0.065%, 0.045%, and 0.063%, suggesting moderate nucleotide-level divergence among *M. sextelata* strains. This diversity may reflect differences in geographic origin, cultivation history, or laboratory domestication, and provides a baseline for future population genomic studies of this commercially important species.

Repetitive sequence annotation revealed that LTR retrotransposons constitute 8.36% of the entire genome and account for 44.09% of all repetitive elements. The distribution of LTR insertion times follows a characteristic “L”-shaped pattern, indicating a recent burst of LTR activity. This suggests that LTR retrotransposon proliferation may have been a key driver of genome expansion in *M. sextelata* [63].

### 4.2. Rapid Genome Remodeling During the Evolution of M. sextelata

Divergence time analysis indicates that *M. sextelata* diverged early within the genus *Morchella*, approximately 14.2 MYA. This divergence sequence is largely consistent with the previous findings [16], although the overall divergence time estimated in this study is relatively more recent. This discrepancy may be attributed to differences in fossil calibration node selection, among other methodological factors. Concurrently, *M. sextelata* exhibits extensive expansion and contraction of gene families. In contrast, significant gene family contraction was observed in a wild morel mushroom *Morchella* sp., whereas the number of expansions was relatively limited [16]. The concurrent pattern of expansion and contraction observed here may reflect the rapid genome remodeling experienced by *M. sextelata* during its evolution, thereby shaping its unique genetic and phenotypic characteristics.

To further investigate the biological functions of rapidly expanded gene families in *M. sextelata*, GO and KEGG pathway enrichment analyses were performed. The results show that these genes are significantly enriched in GO terms such as binding (particularly small molecule, ion, transition metal ion, and protein binding) and secondary metabolite biosynthetic processes. In KEGG pathways, they are significantly enriched in categories including the Fanconi anemia pathway, DNA repair and recombination proteins, and protein phosphatases and their associated proteins. This enrichment pattern suggests two prominent functional themes for the rapid genome remodeling in *M. sextelata*: genes involved in small molecule/ion binding and secondary metabolite biosynthesis, and genes associated with DNA repair and recombination pathways.

The significant enrichment of “small molecule/ion binding” and “secondary metabolite biosynthetic processes” is consistent with expanded capacities for small molecule sensing and secondary metabolite production. Transition metal ions (such as iron, zinc, copper) are key cofactors for many enzymes [64], and the expansion of their binding capacity may optimize cellular metal ion homeostasis and related metabolic efficiency in variable soil environments. Meanwhile, the expansion of secondary metabolic pathways is directly linked to the potential of *M. sextelata* to synthesize novel or specific secondary metabolites. These metabolites may contribute to antimicrobial activity, stress resistance, or interactions with other organisms. However, the specific ecological functions of these predicted metabolites remain to be experimentally determined.

Particularly noteworthy is that KEGG analysis reveals significant enrichment in the Fanconi anemia pathway and DNA repair and recombination proteins. The Fanconi anemia pathway is a core pathway responsible for repairing DNA interstrand crosslinks, and its function is closely related to homologous recombination repair [65,66]. This finding suggests that the rapid genome remodeling process itself may be accompanied by a higher risk of DNA damage, raising the possibility of co-evolution between genome remodeling machinery and DNA repair systems.

### 4.3. Retrotransposons Encoded by Unique Gene Families May Drive Genome Remodeling

To further elucidate the specific mechanisms underlying genome remodeling, this study conducted an in-depth analysis of the unique gene families in *M. sextelata*. In a comparative analysis encompassing 12 fungal species (including *M. sextelata*), a total of 14,866 gene families were identified, of which 2669 were core gene families shared by all species, and 1379 were single-copy orthologous gene families. *M. sextelata* possesses 9138 gene families, including 58 unique gene families (comprising 200 genes). This result provides preliminary insight into the unique genetic architecture that has evolved in this species.

Focusing specifically on intragenus comparisons within *Morchella*, it was found that *M. sextelata* shares 7742 gene families with *M. eximia*, *M. conica*, *M. snyderi*, and *M. importuna*, constituting the “genus-level core genome.” Notably, *M. sextelata* shares an additional 486 gene families with three species other than *M. snyderi*, suggesting a closer phylogenetic relationship or convergent ecological adaptation strategies with these species—a finding further supported by phylogenetic analysis. Importantly, *M. sextelata* possesses 81 unique gene families (comprising 252 genes) at the intragenus level. Functional enrichment analysis of these genes revealed that 79 obtained COG annotations, and as many as 45 genes (56.96%) were enriched in the “replication, recombination, and repair” category, all belonging to the same rapidly expanding gene family.

Domain analysis provided a direct mechanistic explanation for the expansion of this DNA repair system. These genes primarily encode retrotransposon Gag proteins and Ty3 transposon capsid-like proteins, most of which contain zinc finger domains and possess nucleic acid-binding capabilities. Retrotransposons are ubiquitous mobile genetic elements in eukaryotic genomes, and recent studies have increasingly highlighted their role as key drivers of genomic dynamics, evolutionary innovation, and species adaptation [67,68,69]. In *M. sextelata*, these enriched retrotransposon-related genes are not randomly distributed but exist as organized families encoding proteins with specific domains (zinc finger, capsid), suggesting they may have been integrated into the host’s genetic regulatory network.

Thus, the coordinated observation of rapid gene family turnover and DNA repair system expansion in *M. sextelata* raises the hypothesis that transposable element activity could have contributed to genome remodeling, while the expanded DNA repair machinery may have co-evolved as a mechanism to buffer against the resultant genomic instability. Although speculative in the absence of functional validation, the enrichment of retrotransposon-related genes in unique gene families suggests possible roles in processes such as gene network regulation, stress responsiveness, or interspecies signal transduction. This study delineates the genetic positioning of *M. sextelata* within the *Morchella* genus at the genomic level. The abundance of its unique gene families, particularly the specificity of retrotransposon-related gene families, is consistent with the hypothesis that TE-mediated processes have shaped the evolutionary trajectory of this species. This phenomenon resembles cases of “transposon domestication” observed in certain plants or animals [70,71], raising the intriguing possibility that similar mechanisms may operate in fungal genome evolution. Future functional studies, including transposon activity assays and gene expression analyses across developmental stages, will be necessary to test these hypotheses.

### 4.4. The Composition Characteristics of CAZymes Provide Insights into the Predicted Carbohydrate-Degrading Capabilities of M. sextelata

This study further investigated the genetic basis of carbohydrate metabolism in *M. sextelata*. A total of 377 CAZymes were annotated in the *M. sextelata* genome, which is slightly lower than the numbers in *M. crassipes* (409), *M. importuna* (419), and *M. snyderi* (411) [14], but slightly higher than that of a wild morel mushroom (366) [16]. Overall, no significant differences were observed in the total number of CAZymes among these morel species. Regarding the distribution of CAZyme families, *M. sextelata* exhibited the highest number of GHs, followed by AAs and GTs. This compositional pattern is largely consistent with those of *M. crassipes*, *M. importuna*, and *M. snyderi*, with similar quantities across categories. Notably, the number of CBMs in *M. sextelata* was significantly lower than in other *Morchella* species, which may account for its relatively lower total CAZyme count. The marked reduction in CBM numbers may reflect distinct substrate recognition and binding strategies in *M. sextelata* compared to other morels, potentially associated with distinct substrate utilization preferences. However, whether these genomic differences translate into functional distinctions awaits experimental investigation.

Compared with other CAZyme-rich species, GHs generally represent the most abundant enzyme class, yet their specific family composition varies considerably across species. For instance, in *Flammulina elastica*, GH16, GH5, and GH18 are the most abundant GH families [72]; in *Trichoderma harzianum*, GH18, GH3, and GH16 predominate [73]; whereas in *M. sextelata*, GH5, GH43, and GH3 are the most prevalent. This distinct profile suggests a predicted capacity for synergistic degradation of cellulose and hemicellulose: GH5 may cleave the cellulose backbone, GH43 may hydrolyze hemicellulose side chains such as xylan, and GH3 may further break down oligosaccharides into monosaccharides. However, these are in silico predictions based solely on gene content; experimental validation (e.g., enzyme activity assays or transcriptomic profiling under different carbon sources) would be required to confirm these functional assignments. This predicted enzymatic repertoire is compatible with a saprotrophic or symbiotic lifestyle, although the ecological role of *M. sextelata* cannot be conclusively determined from genomic data alone.

### 4.5. M. sextelata May Possess a Large Number of Novel or Highly Differentiated Secondary Metabolic Pathways

In addition to carbohydrate metabolism, the ability to synthesize secondary metabolites represents another important aspect of fungal ecological adaptation. Using antiSMASH analysis, this study identified a total of 152 genes involved in secondary metabolite biosynthesis in the *M. sextelata* genome. These genes are distributed across multiple scaffolds and are organized into 12 BGCs. The BGCs are diverse in type, comprising four terpene synthase clusters, two terpene precursor synthesis clusters, four NRPS-like clusters, and one hybrid cluster involving NRPS, NRPS-like, and T1PKS components. These findings align with previous predictions of secondary metabolite gene clusters in *M. sextelata* [13] and in a wild *Morchella* species [16], both of which reported terpene synthase and T1PKS clusters. However, neither of these earlier studies detected an NRP-siderophore synthesis cluster. This distribution pattern indicates that *M. sextelata* harbors substantial genetic potential for secondary metabolite biosynthesis, with notable phylogenetic specificity in terpenoid and NRPS pathways.

Notably, among the identified BGCs, only Region 11 exhibited approximately 50% sequence similarity to the known clavaric acid biosynthetic gene cluster, whereas the remaining clusters showed no significant matches to functionally characterized BGCs. This suggests that *M. sextelata* may possess numerous novel or highly divergent secondary metabolic pathways; however, the actual products, their structures, and their biological functions remain to be experimentally characterized. The presence of these clusters indicates the genetic potential for producing diverse secondary metabolites. Whether these clusters are actively expressed and what biological roles their products serve await future transcriptomic and metabolomic investigation.

## 5. Conclusions

This study provides a chromosome-level genome of *M. sextelata*. Systematic analysis of this genome reveals that *M. sextelata* has undergone extensive genomic remodeling, coinciding with an enrichment of retrotransposon-related genes and expansion of DNA repair systems. These genomic features co-occur with predicted capabilities in two dimensions: carbohydrate-active enzyme repertoires (CAZymes) suggestive of plant cell wall polysaccharide degradation and biosynthetic gene clusters (secondary metabolism) indicative of terpenoid and non-ribosomal peptide biosynthetic potential. While the correlation between TE enrichment and genomic innovation is striking, future functional studies are needed to establish the causal mechanisms underlying these evolutionary patterns.

## Figures and Tables

**Table 1:** Summary of genome assembly and annotations of *M. sextelata*.

Description	Statistics
Genome size	54,639,260 nt
Pseudochromosomes	26
Scaffold number	33
Scaffold max	4,534,616 nt
Contig number	38
Gap number	5
Scaffold N50	1,942,406 nt
Scaffold L50	10
Contig N50	1,942,406 nt
Contig L50	10
Genes	11,269
GC content	47.38%
Repetitive sequences	18.96%
BUSCO genome integrity	98.8%
BUSCO protein integrity	96.3%

**Table 2:** Comparison of *M. sextelata* genome assemblies. Key assembly metrics are compared between the present study (m-ty1) and three previously published *M. sextelata* genomes. ANI, average nucleotide identity; SNPs, single nucleotide polymorphisms identified by whole-genome alignment; Nucleotide divergence, proportion of mismatched bases over total aligned bases (SNPs/aligned bp × 100). Dashes indicate data not reported in the original publications.

Feature	m-ty1	2019 (Mei et al.) [13]	2021 (GCA_020137385)	2022 (GCA_024713665)
Assembly level	Chromosome	Contig	Contig	Contig
Size (Mb)	54.64	52.93	53.52	53.61
Contig number	38	59	42	28
Contig N50 (Mb)	1.94	1.57	1.82	1.90
Gene number	11,269	9550	13,182	–
ANI vs. m-ty1 (%)	100	99.83	99.86	99.76
SNPs vs. m-ty1	0	29,368	20,604	28,212
Nucleotide divergence (%)	0	0.065	0.045	0.063

**Table 3:** The putative BGCs responsible for secondary metabolites in *M. sextelata*.

Region No.	Gene Cluster Type	Length (nt)	Location (nt)	Scaffold
1	terpene	21,705	2,555,372–2,577,076	scaffold_2
2	terpene	21,672	402,314–423,985	scaffold_6
3	NRPS-like	43,241	1,997,006–2,040,246	scaffold_6
4	NRPS-like	44,050	327,456–371,505	scaffold_9
5	terpene	21,506	1,857,614–1,879,119	scaffold_10
6	NRPS-like	43,416	1,708,134–1,751,549	scaffold_11
7	NRPS-like, T1PKS	53,115	382,445–435,559	scaffold_12
8	terpene-precursor	21,262	880,827–902,088	scaffold_16
9	NRPS-like	43,349	703,630–746,978	scaffold_17
10	terpene-precursor	21,233	333,383–354,615	scaffold_19
11	terpene	22,102	972,559–994,660	scaffold_19
12	NRP-metallophore, NRPS	74,062	560,759–634,820	scaffold_22

## Data Availability

The genome assembly and annotation have been deposited at NCBI GenBank under BioProject PRJNA1458116 and are currently under review. Upon approval, accession numbers will be provided. The raw data has been provided to NCBI with the SRA project ID PRJNA1443680.

## Supplementary Materials

The following supporting information can be downloaded at: https://www.mdpi.com/article/10.3390/jof12050352/s1, Figure S1: Genome-wide Hi-C contact map of *M. sextelata*; Figure S2: Linear relationship between LTR insertion density and insertion time; Figure S3: COG Classification Profile with Logarithmic Transformation of *M. sextelata*; Table S1: Comparative Genomic Overview; Table S2: The gene IDs, gene family classifications, and PFAM domain IDs of genes related to (L) replication, recombination, and repair functions among the unique genes of *M. sextelata*; Table S3: The number of each classification in the Auxiliary Activities (AA) families of CAZymes in *M. sextelata*; Table S4: Genes unique to *M. sextelata* among five *Morchella* species and their predicted Pfam domains and COG categories; Table S5: Genomic locations and gene compositions of predicted biosynthetic gene clusters in *M. sextelata*; Table S6: CAZyme genes annotated in the *M. sextelata* genome.
