# Supplementary material for: Chromosome-Level Genome Assembly of Morchella sextelata Reveals Its Early Divergence and Adaptive Evolution
Source: J Fungi (Basel). 2026 May 10;12(5):352. doi: 10.3390/jof12050352 (PMC13208314; doi:10.3390/jof12050352)
Supplement: Supplementary file 1 [file jof-12-00352-s001.zip › jof-4269212-supplementary.pdf]

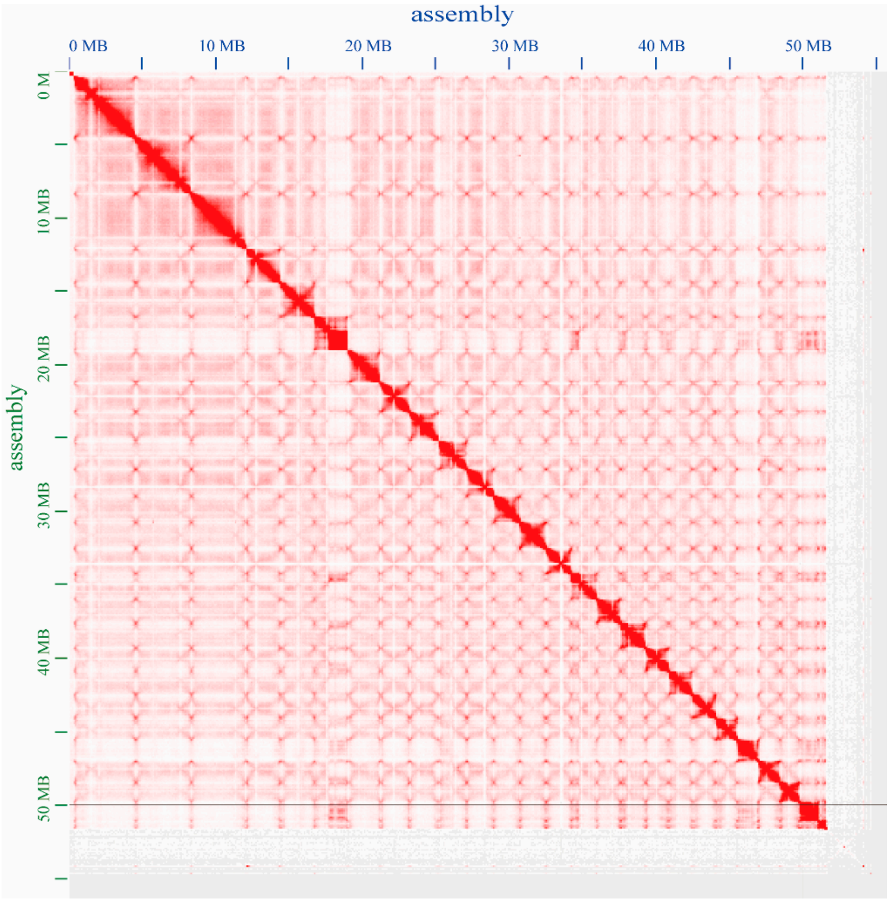


Figure S1: Genome-wide Hi-C contact map of *M. sextelata.* The heatmap shows normalized intra- and inter-chromosomal interaction frequencies at 100 kb resolution. Chromosomes are arranged along both axes, with the color intensity representing the strength of chromatin contacts (darker colors indicate higher interaction frequencies). The diagonal represents interactions within the same chromosome.

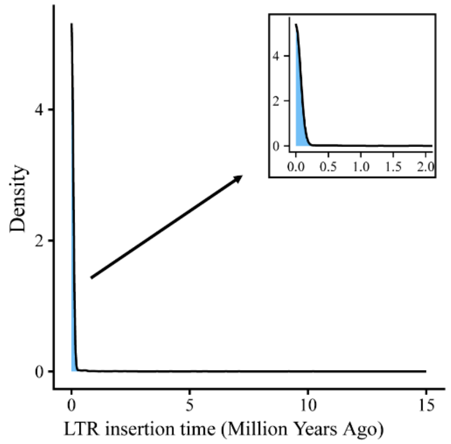


Figure S2: Linear relationship between LTR insertion density and insertion time. The inset panel provides a magnified view of the region near the recent insertion events (x-axis: 0–2 MYA), offering better resolution of the rapid increase in LTR activity toward the present.

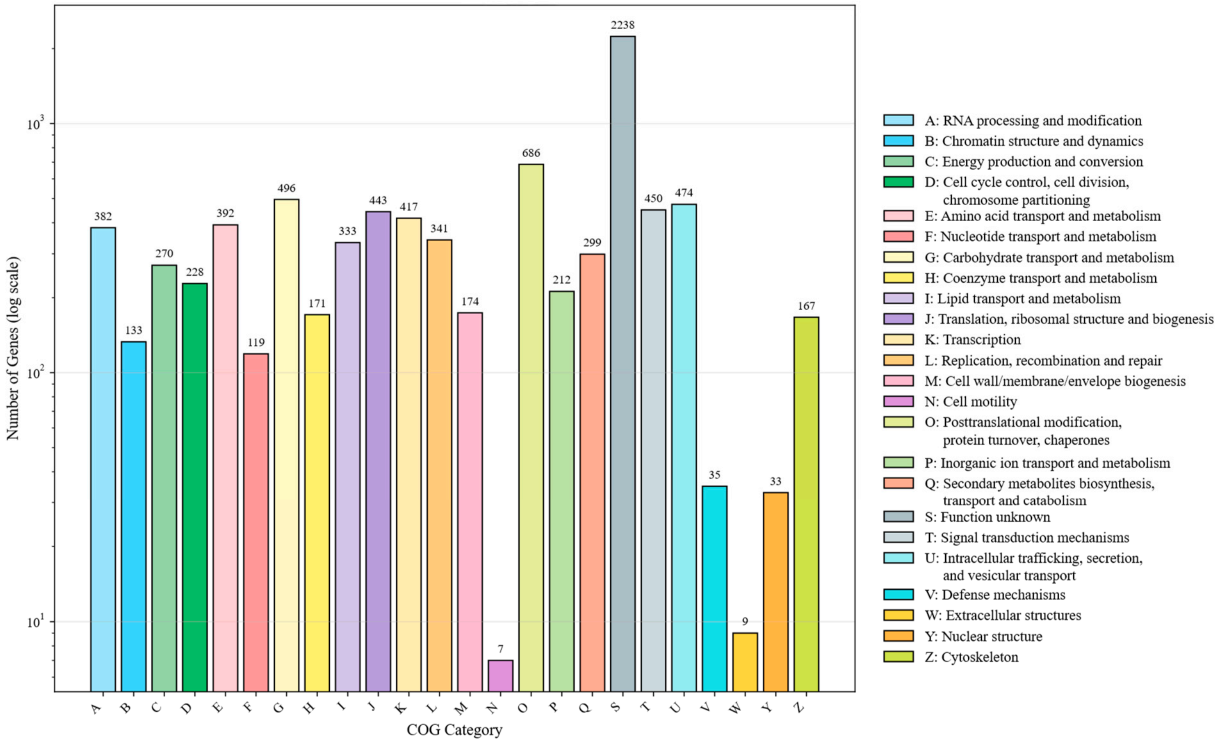


Figure S3: COG Classification Profile with Logarithmic Transformation of *M. sextelata*

Table S1: Comparative Genomic Overview

| Description | Count |
|---|---|
| All gene families | 14,866 |
| All genes | 116,735 |
| Unique orthologous groups | 1,379 |
| All gene families in *M. sextelata* | 9,138 |
| Unique gene families in *M. sextelata* | 58 |
| Unique genes in *M. sextelata* | 200 |

Table S2: The gene IDs, gene family classifications, and PFAM domain IDs of genes related to (L)replication, recombination, and repair functions among the unique genes of *M. sextelata*.

| Gene Family | Gene IDs | COG | PFAM |
|---|---|---|---|
| OG0000054 | AC19I8_000036-T1 | L | PF03732 |
| OG0000054 | AC19I8_000510-T1 | L | PF03732 |
| OG0000054 | AC19I8_000577-T1 | L | PF03732;PF19259 |
| OG0000054 | AC19I8_000605-T1 | L | PF03732;PF19259 |
| OG0000054 | AC19I8_001713-T1 | L | PF03732;PF19259 |
| OG0000054 | AC19I8_002507-T1 | L | PF03732 |
| OG0000054 | AC19I8_003206-T1 | L | PF03732;PF19259 |
| OG0000054 | AC19I8_003366-T1 | L | PF03732;PF19259 |
| OG0000054 | AC19I8_004625-T1 | L | PF03732 |
| OG0000054 | AC19I8_004790-T1 | L | PF03732 |
| OG0000054 | AC19I8_004794-T1 | L | PF03732;PF19259 |
| OG0000054 | AC19I8_004817-T1 | L | PF03732 |
| OG0000054 | AC19I8_005986-T1 | L | PF03732 |
| OG0000054 | AC19I8_006195-T1 | L | PF03732;PF19259 |
| OG0000054 | AC19I8_006217-T1 | L | PF03732;PF19259 |
| OG0000054 | AC19I8_006616-T1 | L | PF03732 |
| OG0000054 | AC19I8_007838-T1 | L | PF03732;PF19259 |
| OG0000054 | AC19I8_008286-T1 | L | PF03732 |
| OG0000054 | AC19I8_008474-T1 | L | PF03732 |
| OG0000054 | AC19I8_008536-T1 | L | PF03732;PF19259 |
| OG0000054 | AC19I8_008693-T1 | L | PF03732;PF19259 |
| OG0000054 | AC19I8_008947-T1 | L | PF03732;PF19259 |
| OG0000054 | AC19I8_009776-T1 | L | PF03732;PF19259 |
| OG0000054 | AC19I8_009873-T1 | L | PF03732 |
| OG0000054 | AC19I8_009976-T1 | L | PF03732 |
| OG0000054 | AC19I8_010524-T1 | L | PF03732 |
| OG0000054 | AC19I8_010537-T1 | L | PF03732 |
| OG0000054 | AC19I8_010593-T1 | L | PF03732 |
| OG0000054 | AC19I8_010609-T1 | L | PF03732;PF19259 |
| OG0000054 | AC19I8_010611-T1 | L | PF03732;PF19259 |
| OG0000054 | AC19I8_010612-T1 | L | PF03732;PF19259 |

| | | | |
|---|---|---|---|
| OG0000054 | AC19I8_010637-T1 | L | PF03732 |
| OG0000054 | AC19I8_010673-T1 | L | PF03732 |
| OG0000054 | AC19I8_011254-T1 | L | PF03732;PF19259 |
| OG0000054 | AC19I8_011305-T1 | L | PF03732;PF19259 |
| OG0000054 | AC19I8_011343-T1 | L | PF03732;PF19259 |
| OG0000054 | AC19I8_011367-T1 | L | PF03732 |
| OG0000054 | AC19I8_011374-T1 | L | PF03732;PF19259 |
| OG0000054 | AC19I8_011379-T1 | L | PF03732 |
| OG0000054 | AC19I8_011408-T1 | L | PF03732;PF19259 |
| OG0000054 | AC19I8_011409-T1 | L | PF03732 |
| OG0000054 | AC19I8_011578-T1 | L | PF03732;PF19259 |
| OG0011333 | AC19I8_000596-T1 | L | |
| OG0013866 | AC19I8_000578-T1 | L | |
| OG0013866 | AC19I8_000597-T1 | L | |

Table S3: The number of each classification in the Auxiliary Activities (AA) families of CAZymes in *M. sextelata.*

| **CAZyme Family** | **Count** |
|---|---|
| AA9 | 21 |
| AA7 | 16 |
| AA3 | 13 |
| AA11 | 9 |
| AA1 | 7 |
| AA14 | 3 |
| AA8 | 3 |
| AA2 | 2 |
| AA12 | 1 |
| AA16 | 1 |
| AA4 | 1 |
| AA5 | 1 |
| AA6 | 1 |

Table S4: Genes unique to *M. sextelata* among five *Morchella* species and their predicted Pfam domains and COG categories.

| **Gene familes** | **GeneID** | **PFAM** | **COG categories** |
|---|---|---|---|
| OG0000002 | AC19I8_010503 | PF05729 | S:(S) Function unknown |
| OG0000015 | AC19I8_010571 | | I:(I) Lipid transport and metabolism;Z:(Z) Cytoskeleton |
| OG0000033 | AC19I8_007402 | PF00023;PF12796; PF13637 | T:(T) Signal transduction mechanisms |
| OG0000052 | AC19I8_010985 | | S:(S) Function unknown |
| OG0000054 | AC19I8_000036 | PF03732 | L:(L) Replication, recombination and repair |
| OG0000054 | AC19I8_000510 | PF03732 | L:(L) Replication, recombination and repair |
| OG0000054 | AC19I8_000577 | PF03732;PF19259 | L:(L) Replication, recombination and repair |
| OG0000054 | AC19I8_000605 | PF03732;PF19259 | L:(L) Replication, recombination and repair |

| | | | |
|---|---|---|---|
| OG0000054 | AC19I8_001713 | PF03732;PF19259 | L:(L) Replication, recombination and repair |
| OG0000054 | AC19I8_002507 | PF03732 | L:(L) Replication, recombination and repair |
| OG0000054 | AC19I8_003206 | PF03732;PF19259 | L:(L) Replication, recombination and repair |
| OG0000054 | AC19I8_003366 | PF03732;PF19259 | L:(L) Replication, recombination and repair |
| OG0000054 | AC19I8_004625 | PF03732 | L:(L) Replication, recombination and repair |
| OG0000054 | AC19I8_004790 | PF03732 | L:(L) Replication, recombination and repair |
| OG0000054 | AC19I8_004794 | PF03732;PF19259 | L:(L) Replication, recombination and repair |
| OG0000054 | AC19I8_004817 | PF03732 | L:(L) Replication, recombination and repair |
| OG0000054 | AC19I8_005986 | PF03732 | L:(L) Replication, recombination and repair |
| OG0000054 | AC19I8_006195 | PF03732;PF19259 | L:(L) Replication, recombination and repair |
| OG0000054 | AC19I8_006217 | PF03732;PF19259 | L:(L) Replication, recombination and repair |
| OG0000054 | AC19I8_006616 | PF03732 | L:(L) Replication, recombination and repair |
| OG0000054 | AC19I8_007838 | PF03732;PF19259 | L:(L) Replication, recombination and repair |
| OG0000054 | AC19I8_008283 | PF03732 | |
| OG0000054 | AC19I8_008286 | PF03732 | L:(L) Replication, recombination and repair |
| OG0000054 | AC19I8_008474 | PF03732 | L:(L) Replication, recombination and repair |
| OG0000054 | AC19I8_008536 | PF03732;PF19259 | L:(L) Replication, recombination and repair |
| OG0000054 | AC19I8_008693 | PF03732;PF19259 | L:(L) Replication, recombination and repair |
| OG0000054 | AC19I8_008947 | PF03732;PF19259 | L:(L) Replication, recombination and repair |
| OG0000054 | AC19I8_008956 | PF03732 | |
| OG0000054 | AC19I8_009776 | PF03732;PF19259 | L:(L) Replication, recombination and repair |
| OG0000054 | AC19I8_009873 | PF03732 | L:(L) Replication, recombination and repair |
| OG0000054 | AC19I8_009976 | PF03732 | L:(L) Replication, recombination and repair |
| OG0000054 | AC19I8_010524 | PF03732 | L:(L) Replication, recombination and repair |
| OG0000054 | AC19I8_010537 | PF03732 | L:(L) Replication, recombination and repair |
| OG0000054 | AC19I8_010593 | PF03732 | L:(L) Replication, recombination and repair |
| OG0000054 | AC19I8_010609 | PF03732;PF19259 | L:(L) Replication, recombination and repair |
| OG0000054 | AC19I8_010611 | PF03732;PF19259 | L:(L) Replication, recombination and repair |
| OG0000054 | AC19I8_010612 | PF03732;PF19259 | L:(L) Replication, recombination and repair |
| OG0000054 | AC19I8_010637 | PF03732 | L:(L) Replication, recombination and repair |
| OG0000054 | AC19I8_010658 | | |
| OG0000054 | AC19I8_010673 | PF03732 | L:(L) Replication, recombination and repair |
| OG0000054 | AC19I8_011254 | PF03732;PF19259 | L:(L) Replication, recombination and repair |
| OG0000054 | AC19I8_011305 | PF03732;PF19259 | L:(L) Replication, recombination and repair |
| OG0000054 | AC19I8_011343 | PF03732;PF19259 | L:(L) Replication, recombination and repair |
| OG0000054 | AC19I8_011367 | PF03732 | L:(L) Replication, recombination and repair |
| OG0000054 | AC19I8_011374 | PF03732;PF19259 | L:(L) Replication, recombination and repair |
| OG0000054 | AC19I8_011379 | PF03732 | L:(L) Replication, recombination and repair |
| OG0000054 | AC19I8_011408 | PF03732;PF19259 | L:(L) Replication, recombination and repair |
| OG0000054 | AC19I8_011409 | PF03732 | L:(L) Replication, recombination and repair |
| OG0000054 | AC19I8_011524 | PF03732 | S:(S) Function unknown |
| OG0000054 | AC19I8_011578 | PF03732;PF19259 | L:(L) Replication, recombination and repair |
| OG0000254 | AC19I8_010659 | | |
| OG0000254 | AC19I8_010660 | | |
| OG0000254 | AC19I8_010661 | | |
| OG0000254 | AC19I8_011605 | | |
| OG0000254 | AC19I8_011608 | | |
| OG0000254 | AC19I8_011610 | | |
| OG0000254 | AC19I8_011612 | | |
| OG0000254 | AC19I8_011614 | | |
| OG0000254 | AC19I8_011616 | | |
| OG0000254 | AC19I8_011621 | | |
| OG0000254 | AC19I8_011623 | | |
| OG0000254 | AC19I8_011625 | | |
| OG0000254 | AC19I8_011627 | | |
| OG0000254 | AC19I8_011629 | | |
| OG0000254 | AC19I8_011630 | | |
| OG0000254 | AC19I8_011632 | | |
| OG0000254 | AC19I8_011634 | | |
| OG0000254 | AC19I8_011638 | | |

| | | | |
|---|---|---|---|
| OG0000254 | AC19I8_011640 | | |
| OG0000254 | AC19I8_011642 | | |
| OG0000254 | AC19I8_011644 | | |
| OG0000254 | AC19I8_011647 | | |
| OG0000254 | AC19I8_011650 | | |
| OG0000254 | AC19I8_011652 | | |
| OG0000254 | AC19I8_011654 | | |
| OG0000312 | AC19I8_011604 | | |
| OG0000312 | AC19I8_011607 | | |
| OG0000312 | AC19I8_011609 | | |
| OG0000312 | AC19I8_011611 | | |
| OG0000312 | AC19I8_011613 | | |
| OG0000312 | AC19I8_011615 | | |
| OG0000312 | AC19I8_011618 | | |
| OG0000312 | AC19I8_011620 | | |
| OG0000312 | AC19I8_011622 | | |
| OG0000312 | AC19I8_011624 | | |
| OG0000312 | AC19I8_011626 | | |
| OG0000312 | AC19I8_011628 | | |
| OG0000312 | AC19I8_011631 | | |
| OG0000312 | AC19I8_011633 | | |
| OG0000312 | AC19I8_011637 | | |
| OG0000312 | AC19I8_011639 | | |
| OG0000312 | AC19I8_011641 | | |
| OG0000312 | AC19I8_011643 | | |
| OG0000312 | AC19I8_011645 | | |
| OG0000312 | AC19I8_011649 | | |
| OG0000312 | AC19I8_011651 | | |
| OG0000312 | AC19I8_011653 | | |
| OG0000586 | AC19I8_005767 | | |
| OG0001475 | AC19I8_005364 | | S:(S) Function unknown |
| OG0002332 | AC19I8_002508 | PF00082 | S:(S) Function unknown |
| OG0002332 | AC19I8_002512 | PF00082 | S:(S) Function unknown |
| OG0003879 | AC19I8_007012 | PF00004;PF17866 | O:(O) Posttranslational modification, protein turnover, chaperones |
| OG0006340 | AC19I8_009903 | PF14441 | S:(S) Function unknown |
| OG0006607 | AC19I8_005995 | | |
| OG0007026 | AC19I8_000575 | | |
| OG0007026 | AC19I8_010615 | | |
| OG0007026 | AC19I8_010616 | | |
| OG0007026 | AC19I8_010618 | | |
| OG0007026 | AC19I8_010619 | | |
| OG0007026 | AC19I8_010620 | | |
| OG0007026 | AC19I8_011580 | | |
| OG0007027 | AC19I8_004885 | PF12511 | S:(S) Function unknown |
| OG0007027 | AC19I8_005542 | PF12511 | S:(S) Function unknown |
| OG0007027 | AC19I8_006881 | PF12511 | S:(S) Function unknown |
| OG0007027 | AC19I8_007847 | PF12511 | S:(S) Function unknown |
| OG0007027 | AC19I8_008539 | PF12511 | S:(S) Function unknown |
| OG0007164 | AC19I8_008250 | | |
| OG0007229 | AC19I8_004670 | | S:(S) Function unknown |
| OG0007865 | AC19I8_002511 | | |
| OG0009251 | AC19I8_008897 | PF00023;PF12796; PF13606;PF13637; PF13857 | S:(S) Function unknown |
| OG0009453 | AC19I8_006818 | | |
| OG0009466 | AC19I8_000306 | | Q:(Q) Secondary metabolites biosynthesis, transport and catabolism |
| OG0010076 | AC19I8_001607 | | |
| OG0010076 | AC19I8_004616 | PF12776 | |

| | | | |
|---|---|---|---|
| OG0010076 | AC19I8_005677 | PF12776 | S:(S) Function unknown |
| OG0010076 | AC19I8_009930 | | |
| OG0010077 | AC19I8_001933 | | |
| OG0010077 | AC19I8_002766 | | |
| OG0010077 | AC19I8_002767 | | |
| OG0010077 | AC19I8_010642 | | |
| OG0010078 | AC19I8_004369 | | |
| OG0010078 | AC19I8_005539 | | |
| OG0010078 | AC19I8_008541 | | |
| OG0010078 | AC19I8_011534 | | |
| OG0010083 | AC19I8_007013 | | |
| OG0010083 | AC19I8_007014 | | |
| OG0010083 | AC19I8_010883 | | |
| OG0010330 | AC19I8_000547 | | |
| OG0010330 | AC19I8_011667 | | |
| OG0011330 | AC19I8_000005 | | |
| OG0011330 | AC19I8_000006 | PF03732 | |
| OG0011330 | AC19I8_000538 | PF03732 | |
| OG0011332 | AC19I8_000140 | | |
| OG0011332 | AC19I8_000407 | | |
| OG0011332 | AC19I8_000407 | | |
| OG0011333 | AC19I8_000596 | | L:(L) Replication, recombination and repair |
| OG0011337 | AC19I8_003870 | | |
| OG0011337 | AC19I8_004368 | | |
| OG0011337 | AC19I8_008902 | | |
| OG0011340 | AC19I8_007842 | | |
| OG0011340 | AC19I8_008922 | | |
| OG0011340 | AC19I8_010302 | | |
| OG0011341 | AC19I8_007982 | | |
| OG0011341 | AC19I8_007982 | | |
| OG0011341 | AC19I8_007982 | | |
| OG0011345 | AC19I8_011606 | | |
| OG0011345 | AC19I8_011617 | | |
| OG0011345 | AC19I8_011648 | | |
| OG0013845 | AC19I8_000003 | | |
| OG0013845 | AC19I8_000540 | | |
| OG0013846 | AC19I8_000004 | | |
| OG0013846 | AC19I8_000539 | | |
| OG0013847 | AC19I8_000008 | | |
| OG0013847 | AC19I8_000536 | | |
| OG0013848 | AC19I8_000017 | | |
| OG0013848 | AC19I8_000528 | | |
| OG0013849 | AC19I8_000018 | | |
| OG0013849 | AC19I8_000527 | | |
| OG0013850 | AC19I8_000025 | | |
| OG0013850 | AC19I8_000521 | | |
| OG0013851 | AC19I8_000054 | | |
| OG0013851 | AC19I8_000494 | | |
| OG0013852 | AC19I8_000084 | PF00023;PF12796 | Z:(Z) Cytoskeleton |
| OG0013852 | AC19I8_000465 | PF00023;PF12796 | Z:(Z) Cytoskeleton |
| OG0013853 | AC19I8_000102 | | |
| OG0013853 | AC19I8_000448 | | |
| OG0013854 | AC19I8_000205 | | |
| OG0013854 | AC19I8_000347 | | |
| OG0013855 | AC19I8_000228 | | |
| OG0013855 | AC19I8_000325 | | |
| OG0013856 | AC19I8_000229 | | |
| OG0013856 | AC19I8_000324 | | |
| OG0013857 | AC19I8_000231 | | |
| OG0013857 | AC19I8_000322 | | |

| | | | |
|---|---|---|---|
| OG0013858 | AC19I8_000239 | | |
| OG0013858 | AC19I8_000314 | | |
| OG0013860 | AC19I8_000543 | | |
| OG0013860 | AC19I8_011670 | | |
| OG0013861 | AC19I8_000544 | | |
| OG0013861 | AC19I8_011669 | | |
| OG0013862 | AC19I8_000564 | PF12770 | O:(O) Posttranslational modification, protein turnover, chaperones |
| OG0013862 | AC19I8_011590 | PF12770 | O:(O) Posttranslational modification, protein turnover, chaperones |
| OG0013863 | AC19I8_000565 | | |
| OG0013863 | AC19I8_011589 | | |
| OG0013864 | AC19I8_000574 | | |
| OG0013864 | AC19I8_011581 | | |
| OG0013865 | AC19I8_000576 | | |
| OG0013865 | AC19I8_011579 | | |
| OG0013866 | AC19I8_000578 | | L:(L) Replication, recombination and repair |
| OG0013866 | AC19I8_000597 | | L:(L) Replication, recombination and repair |
| OG0013867 | AC19I8_000580 | | |
| OG0013867 | AC19I8_000599 | | |
| OG0013871 | AC19I8_001602 | | |
| OG0013871 | AC19I8_009918 | | |
| OG0013873 | AC19I8_002266 | | |
| OG0013873 | AC19I8_009415 | | |
| OG0013876 | AC19I8_002755 | | |
| OG0013876 | AC19I8_002755 | | |
| OG0013880 | AC19I8_003430 | | |
| OG0013880 | AC19I8_003430 | | |
| OG0013882 | AC19I8_003488 | | |
| OG0013882 | AC19I8_007634 | | |
| OG0013888 | AC19I8_004663 | | |
| OG0013888 | AC19I8_011296 | | |
| OG0013889 | AC19I8_004875 | | |
| OG0013890 | AC19I8_004898 | | |
| OG0013890 | AC19I8_006443 | | |
| OG0013892 | AC19I8_005270 | | |
| OG0013892 | AC19I8_005276 | | |
| OG0013896 | AC19I8_005779 | PF00023;PF00069; PF07714;PF12796; PF13606;PF13637; PF13857 | D:(D) Cell cycle control, cell division, chromosome partitioning |
| OG0013896 | AC19I8_005779 | PF00023;PF00069; PF07714;PF12796; PF13606;PF13637; PF13857 | T:(T) Signal transduction mechanisms |
| OG0013899 | AC19I8_005994 | | |
| OG0013899 | AC19I8_009996 | | |
| OG0013908 | AC19I8_006663 | PF13359 | S:(S) Function unknown |
| OG0013908 | AC19I8_007623 | PF13359 | S:(S) Function unknown |
| OG0013913 | AC19I8_008120 | | |
| OG0013915 | AC19I8_008537 | | |
| OG0013915 | AC19I8_010664 | | |
| OG0013916 | AC19I8_008696 | | S:(S) Function unknown |
| OG0013916 | AC19I8_008696 | | S:(S) Function unknown |
| OG0013917 | AC19I8_008901 | | M:(M) Cell wall/membrane/envelope biogenesis |
| OG0013917 | AC19I8_011545 | | K:(K) Transcription;L:(L) Replication, recombination and repair;T:(T) Signal transduction mechanisms |
| OG0013919 | AC19I8_009381 | | |

| | | | |
|---|---|---|---|
| OG0013919 | AC19I8_009381 | | |
| OG0013922 | AC19I8_009623 | PF00023;PF12796; PF13637 | M:(M) Cell wall/membrane/envelope biogenesis |
| OG0013922 | AC19I8_011427 | | |
| OG0013924 | AC19I8_010207 | | |
| OG0013924 | AC19I8_010594 | | |
| OG0013929 | AC19I8_010652 | | |
| OG0013929 | AC19I8_010652 | | |
| OG0013930 | AC19I8_010662 | | |
| OG0013930 | AC19I8_011646 | | |
| OG0013932 | AC19I8_010900 | | |
| OG0013932 | AC19I8_010901 | | |
| OG0013934 | AC19I8_011053 | PF00023;PF12796; PF13606;PF13637 | M:(M) Cell wall/membrane/envelope biogenesis |
| OG0013934 | AC19I8_011053 | PF00023;PF12796; PF13606;PF13637 | M:(M) Cell wall/membrane/envelope biogenesis |
| OG0013936 | AC19I8_011364 | | |
| OG0013936 | AC19I8_011365 | | |
| OG0013937 | AC19I8_011549 | | |
| OG0013937 | AC19I8_011552 | | |
| OG0013938 | AC19I8_011695 | | |
| OG0013938 | AC19I8_011695 | | |

Table S5: Genomic locations and gene compositions of predicted biosynthetic gene clusters in *M. sextelata*.

| **Gene Cluster Type** | **Scaffold** | **Location** | **Genes** |
|---|---|---|---|
| terpene | scaffold_2 | 2,555,372 - 2,577,076 | AC19I8_002214<br>AC19I8_002215<br>AC19I8_002216<br>AC19I8_002217<br>AC19I8_002218 |
| terpene | scaffold_6 | 402,314 - 423,985 | AC19I8_003951<br>AC19I8_003952<br>AC19I8_003953<br>AC19I8_003954<br>AC19I8_003955 |
| NRPS-like | scaffold_6 | 1,997,006 - 2,040,246 | AC19I8_004292<br>AC19I8_004293<br>AC19I8_004294<br>AC19I8_004295<br>AC19I8_004296<br>AC19I8_004297<br>AC19I8_004298<br>AC19I8_004299<br>AC19I8_004300<br>AC19I8_004301 |
| NRPS-like | scaffold_9 | 327,456 - 371,505 | AC19I8_005422<br>AC19I8_005423<br>AC19I8_005424<br>AC19I8_005425<br>AC19I8_005426<br>AC19I8_005427<br>AC19I8_005428 |
| terpene | scaffold_10 | 1,857,614 - 1,879,119 | AC19I8_006204<br>AC19I8_006205<br>AC19I8_006206<br>AC19I8_006207 |

| | | | |
|---|---|---|---|
| NRPS-like | scaffold_11 | 1,708,134 - 1,751,549 | AC19I8_006585<br>AC19I8_006586<br>AC19I8_006587<br>AC19I8_006588<br>AC19I8_006589<br>AC19I8_006590<br>AC19I8_006591 |
| NRPS-like,T1PKS | scaffold_12 | 382,445 - 435,559 | AC19I8_006705<br>AC19I8_006706<br>AC19I8_006707<br>AC19I8_006708<br>AC19I8_006709<br>AC19I8_006710<br>AC19I8_006711<br>AC19I8_006712<br>AC19I8_006713 |
| terpene-precursor | scaffold_16 | 880,827 - 902,088 | AC19I8_008354<br>AC19I8_008356<br>AC19I8_008357<br>AC19I8_008358<br>AC19I8_008359 |
| NRPS-like | scaffold_17 | 703,630 - 746,978 | AC19I8_008696<br>AC19I8_008697<br>AC19I8_008698<br>AC19I8_008699<br>AC19I8_008700<br>AC19I8_008701<br>AC19I8_008702<br>AC19I8_008703<br>AC19I8_008704<br>AC19I8_008705<br>AC19I8_008706<br>AC19I8_008707<br>AC19I8_008708<br>AC19I8_008709<br>AC19I8_008710 |
| terpene-precursor | scaffold_19 | 333,383 - 354,615 | AC19I8_009329<br>AC19I8_009330<br>AC19I8_009331<br>AC19I8_009332<br>AC19I8_009333<br>AC19I8_009334 |
| terpene | scaffold_19 | 972,559 - 994,660 | AC19I8_009479<br>AC19I8_009480<br>AC19I8_009481<br>AC19I8_009482<br>AC19I8_009483 |
| NRP-metallophore, NRPS | scaffold_22 | 560,759 - 634,820 | AC19I8_010305<br>AC19I8_010306<br>AC19I8_010307<br>AC19I8_010308<br>AC19I8_010309<br>AC19I8_010310<br>AC19I8_010311<br>AC19I8_010312<br>AC19I8_010313<br>AC19I8_010314<br>AC19I8_010315<br>AC19I8_010317 |

Table S6: CAZyme genes annotated in the *M. sextelata* genome.

| Gene ID | CAZyme Family | Class |
|---|---|---|
| AC19I8_000063 | GH95 | GH |
| AC19I8_000109 | GH5 | GH |
| AC19I8_000151 | GT20 | GT |
| AC19I8_000198 | AA2 | AA |
| AC19I8_000243 | CE3 | CE |
| AC19I8_000253 | AA3 | AA |
| AC19I8_000299 | AA3 | AA |
| AC19I8_000310 | CE3 | CE |
| AC19I8_000353 | AA2 | AA |
| AC19I8_000395 | GT20 | GT |
| AC19I8_000395 | GT20 | GT |
| AC19I8_000438 | GH5 | GH |
| AC19I8_000485 | GH95 | GH |
| AC19I8_000560 | GH5 | GH |
| AC19I8_000602 | AA7 | AA |
| AC19I8_000620 | GH43 | GH |
| AC19I8_000644 | GT62 | GT |
| AC19I8_000669 | GH72 | GH |
| AC19I8_000683 | GH140 | GH |
| AC19I8_000684 | GT4 | GT |
| AC19I8_000685 | GT4 | GT |
| AC19I8_000688 | GH16 | GH |
| AC19I8_000689 | AA9 | AA |
| AC19I8_000743 | GH6 | GH |
| AC19I8_000747 | GH74 | GH |
| AC19I8_000766 | AA9 | AA |
| AC19I8_000789 | GT8 | GT |
| AC19I8_000831 | AA3 | AA |
| AC19I8_000831 | AA8 | AA |
| AC19I8_000976 | PL4 | PL |
| AC19I8_001001 | AA3 | AA |
| AC19I8_001001 | AA8 | AA |
| AC19I8_001051 | PL1 | PL |
| AC19I8_001052 | CE8 | CE |
| AC19I8_001069 | AA1 | AA |
| AC19I8_001081 | PL1 | PL |
| AC19I8_001081 | PL1 | PL |
| AC19I8_001099 | CE4 | CE |
| AC19I8_001117 | GH51 | GH |
| AC19I8_001125 | CE4 | CE |
| AC19I8_001126 | AA1 | AA |
| AC19I8_001157 | CE1 | CE |
| AC19I8_001213 | GH35 | GH |
| AC19I8_001265 | GT2_Chitin_synth | GT |
| AC19I8_001266 | GT2_Chitin_synth | GT |
| AC19I8_001269 | GH5 | GH |
| AC19I8_001272 | GH81 | GH |
| AC19I8_001274 | GT90 | GT |
| AC19I8_001281 | GH128 | GH |
| AC19I8_001285 | PL3 | PL |
| AC19I8_001354 | GH125 | GH |
| AC19I8_001385 | GT62 | GT |
| AC19I8_001452 | GT35 | GT |
| AC19I8_001512 | GH16 | GH |
| AC19I8_001558 | PL4 | PL |
| AC19I8_001625 | GT8 | GT |
| AC19I8_001647 | GH16 | GH |
| AC19I8_001667 | AA9 | AA |
| AC19I8_001766 | GH134 | GH |
| AC19I8_001770 | GH5 | GH |
| AC19I8_001776 | AA11 | AA |
| AC19I8_001785 | AA5 | AA |
| AC19I8_001785 | CBM18 | CB |
| AC19I8_001796 | GT2_Glycos_transf | GT |
| AC19I8_001839 | AA7 | AA |
| AC19I8_001864 | GT34 | GT |
| AC19I8_002006 | CE16 | CE |
| AC19I8_002015 | GH3 | GH |
| AC19I8_002020 | GH146 | GH |
| AC19I8_002026 | GH88 | GH |
| AC19I8_002031 | GH27 | GH |
| AC19I8_002036 | GH5 | GH |
| AC19I8_002067 | GH5 | GH |
| AC19I8_002173 | AA8 | AA |
| AC19I8_002208 | GH76 | GH |
| AC19I8_002208 | GH76 | GH |
| AC19I8_002250 | GT8 | GT |
| AC19I8_002255 | GH28 | GH |
| AC19I8_002263 | AA14 | AA |
| AC19I8_002338 | GH17 | GH |
| AC19I8_002370 | GT57 | GT |
| AC19I8_002370 | GT57 | GT |
| AC19I8_002379 | GT2_Chitin_synth | GT |
| AC19I8_002384 | GT2_Chitin_synth | GT |
| AC19I8_002460 | GT1 | GT |
| AC19I8_002496 | GH5 | GH |
| AC19I8_002527 | AA9 | AA |
| AC19I8_002541 | CE4 | CE |
| AC19I8_002553 | GH3 | GH |
| AC19I8_002578 | GT69 | GT |
| AC19I8_002593 | GH2 | GH |
| AC19I8_002595 | PL4 | PL |
| AC19I8_002596 | AA9 | AA |
| AC19I8_002598 | AA9 | AA |

| | | | | | |
|---|---|---|---|---|---|
| AC19I8_002632 | CE12 | CE | AC19I8_003748 | GH28 | GH |
| AC19I8_002636 | AA1 | AA | AC19I8_003763 | GH10 | GH |
| AC19I8_002649 | CE5 | CE | AC19I8_003787 | GH26 | GH |
| AC19I8_002658 | GT2_Chitin_synth | GT | AC19I8_003787 | CBM35 | CB |
| AC19I8_002723 | GH16 | GH | AC19I8_003794 | GH85 | GH |
| AC19I8_002724 | GT2_Glyco_trans_2 | GT | AC19I8_003815 | GH16 | GH |
| AC19I8_002726 | GH3 | GH | AC19I8_003922 | GT90 | GT |
| AC19I8_002761 | GH31 | GH | AC19I8_003957 | GT8 | GT |
| AC19I8_002762 | GH13 | GH | AC19I8_003959 | GT62 | GT |
| AC19I8_002799 | AA11 | AA | AC19I8_003961 | GH45 | GH |
| AC19I8_002807 | GH47 | GH | AC19I8_004103 | GH132 | GH |
| AC19I8_002817 | GH15 | GH | AC19I8_004120 | CE8 | CE |
| AC19I8_002838 | GH16 | GH | AC19I8_004132 | AA9 | AA |
| AC19I8_002851 | AA11 | AA | AC19I8_004135 | AA3 | AA |
| AC19I8_002871 | GH76 | GH | AC19I8_004136 | AA7 | AA |
| AC19I8_002926 | PL3 | PL | AC19I8_004138 | GH75 | GH |
| AC19I8_002936 | GH10 | GH | AC19I8_004163 | GH75 | GH |
| AC19I8_002936 | GH10 | GH | AC19I8_004190 | GT39 | GT |
| AC19I8_002936 | GH10 | GH | AC19I8_004273 | GT90 | GT |
| AC19I8_002944 | GH76 | GH | AC19I8_004275 | GH109 | GH |
| AC19I8_002944 | GH76 | GH | AC19I8_004342 | GH12 | GH |
| AC19I8_003119 | AA7 | AA | AC19I8_004354 | GH7 | GH |
| AC19I8_003161 | GH152 | GH | AC19I8_004362 | CE4 | CE |
| AC19I8_003170 | AA1 | AA | AC19I8_004371 | GH26 | GH |
| AC19I8_003179 | GH131 | GH | AC19I8_004371 | CBM35 | CB |
| AC19I8_003223 | GH16 | GH | AC19I8_004392 | CE4 | CE |
| AC19I8_003259 | GH20 | GH | AC19I8_004395 | GH13 | GH |
| AC19I8_003307 | AA3 | AA | AC19I8_004395 | GH13 | GH |
| AC19I8_003315 | GH35 | GH | AC19I8_004397 | GH13 | GH |
| AC19I8_003348 | GH154 | GH | AC19I8_004397 | GH13 | GH |
| AC19I8_003355 | AA14 | AA | AC19I8_004401 | AA9 | AA |
| AC19I8_003378 | GH78 | GH | AC19I8_004513 | PL1 | PL |
| AC19I8_003398 | GH43 | GH | AC19I8_004530 | GH133 | GH |
| AC19I8_003405 | GH6 | GH | AC19I8_004540 | GH47 | GH |
| AC19I8_003472 | GH43 | GH | AC19I8_004660 | GH5 | GH |
| AC19I8_003475 | GH92 | GH | AC19I8_004672 | AA9 | AA |
| AC19I8_003479 | CE4 | CE | AC19I8_004687 | PL1 | PL |
| AC19I8_003486 | PL4 | PL | AC19I8_004704 | GH17 | GH |
| AC19I8_003505 | CE5 | CE | AC19I8_004882 | GH35 | GH |
| AC19I8_003519 | AA9 | AA | AC19I8_004886 | AA7 | AA |
| AC19I8_003559 | GH93 | GH | AC19I8_004890 | AA9 | AA |
| AC19I8_003574 | GH37 | GH | AC19I8_004899 | GH71 | GH |
| AC19I8_003595 | GH6 | GH | AC19I8_004905 | AA11 | AA |
| AC19I8_003595 | GH6 | GH | AC19I8_005033 | AA7 | AA |
| AC19I8_003665 | GT33 | GT | AC19I8_005102 | GH31 | GH |
| AC19I8_003669 | GH5 | GH | AC19I8_005178 | AA3 | AA |
| AC19I8_003733 | AA16 | AA | AC19I8_005198 | GH28 | GH |
| AC19I8_003734 | AA9 | AA | AC19I8_005222 | AA11 | AA |
| AC19I8_003744 | PL1 | PL | AC19I8_005272 | PL1 | PL |
| AC19I8_003747 | AA9 | AA | AC19I8_005295 | AA9 | AA |

| | | | | | |
|---|---|---|---|---|---|
| AC19I8_005296 | CBM52 | CB | AC19I8_006540 | AA1 | AA |
| AC19I8_005328 | CE16 | CE | AC19I8_006549 | GH18 | GH |
| AC19I8_005340 | GT15 | GT | AC19I8_006578 | GH5 | GH |
| AC19I8_005354 | CE12 | CE | AC19I8_006584 | AA7 | AA |
| AC19I8_005361 | GH5 | GH | AC19I8_006600 | GH76 | GH |
| AC19I8_005387 | GH38 | GH | AC19I8_006643 | GT34 | GT |
| AC19I8_005434 | GH43 | GH | AC19I8_006669 | GT15 | GT |
| AC19I8_005462 | GH5 | GH | AC19I8_006701 | GH10 | GH |
| AC19I8_005469 | CE4 | CE | AC19I8_006702 | AA9 | AA |
| AC19I8_005469 | CBM18 | CB | AC19I8_006819 | GT57 | GT |
| AC19I8_005469 | CE4 | CE | AC19I8_006827 | GT39 | GT |
| AC19I8_005469 | CBM18 | CB | AC19I8_006868 | GH74 | GH |
| AC19I8_005506 | GT22 | GT | AC19I8_006914 | CE5 | CE |
| AC19I8_005522 | GH5 | GH | AC19I8_006999 | GH43 | GH |
| AC19I8_005592 | GT32 | GT | AC19I8_007015 | GT69 | GT |
| AC19I8_005662 | GH3 | GH | AC19I8_007020 | GH105 | GH |
| AC19I8_005662 | GH3 | GH | AC19I8_007022 | GH3 | GH |
| AC19I8_005727 | GH43 | GH | AC19I8_007033 | GH127 | GH |
| AC19I8_005731 | GH55 | GH | AC19I8_007033 | GH146 | GH |
| AC19I8_005780 | AA3 | AA | AC19I8_007081 | GH13 | GH |
| AC19I8_005817 | GT20 | GT | AC19I8_007181 | PL1 | PL |
| AC19I8_005829 | GH3 | GH | AC19I8_007182 | PL1 | PL |
| AC19I8_005830 | GH43 | GH | AC19I8_007273 | GH47 | GH |
| AC19I8_005831 | CE5 | CE | AC19I8_007323 | GT2_Chitin_synth | GT |
| AC19I8_005835 | GH115 | GH | AC19I8_007356 | GH28 | GH |
| AC19I8_005836 | GH43 | GH | AC19I8_007421 | CE5 | CE |
| AC19I8_005842 | GH3 | GH | AC19I8_007422 | PL3 | PL |
| AC19I8_005885 | GT20 | GT | AC19I8_007438 | AA7 | AA |
| AC19I8_005948 | GH5 | GH | AC19I8_007498 | GH51 | GH |
| AC19I8_005957 | GT24 | GT | AC19I8_007542 | GH47 | GH |
| AC19I8_005965 | PL42 | PL | AC19I8_007542 | GH47 | GH |
| AC19I8_006062 | GH72 | GH | AC19I8_007572 | GH76 | GH |
| AC19I8_006062 | CBM43 | CB | AC19I8_007614 | GT4 | GT |
| AC19I8_006062 | GH72 | GH | AC19I8_007614 | GT4 | GT |
| AC19I8_006062 | CBM43 | CB | AC19I8_007614 | GT4 | GT |
| AC19I8_006067 | GT1 | GT | AC19I8_007636 | GT76 | GT |
| AC19I8_006067 | GT1 | GT | AC19I8_007662 | GT50 | GT |
| AC19I8_006068 | GH17 | GH | AC19I8_007681 | GT4 | GT |
| AC19I8_006102 | GH18 | GH | AC19I8_007694 | GT22 | GT |
| AC19I8_006111 | GH47 | GH | AC19I8_007769 | GT39 | GT |
| AC19I8_006115 | GH13 | GH | AC19I8_007793 | AA9 | AA |
| AC19I8_006232 | GH43 | GH | AC19I8_007797 | GH5 | GH |
| AC19I8_006236 | GH3 | GH | AC19I8_007817 | GH72 | GH |
| AC19I8_006242 | GH79 | GH | AC19I8_007837 | GH31 | GH |
| AC19I8_006244 | PL3 | PL | AC19I8_007868 | AA7 | AA |
| AC19I8_006250 | GH31 | GH | AC19I8_007889 | AA4 | AA |
| AC19I8_006259 | AA11 | AA | AC19I8_007889 | AA7 | AA |
| AC19I8_006298 | GH17 | GH | AC19I8_007916 | GH95 | GH |
| AC19I8_006413 | GH16 | GH | AC19I8_007920 | GH2 | GH |
| AC19I8_006528 | AA6 | AA | AC19I8_007921 | GH1 | GH |

| | | |
|---|---|---|
| AC19I8_007938 | PL1 | PL |
| AC19I8_008069 | GH16 | GH |
| AC19I8_008128 | AA11 | AA |
| AC19I8_008151 | AA3 | AA |
| AC19I8_008186 | PL4 | PL |
| AC19I8_008244 | AA14 | AA |
| AC19I8_008315 | GH105 | GH |
| AC19I8_008315 | GH105 | GH |
| AC19I8_008334 | GH31 | GH |
| AC19I8_008420 | GT8 | GT |
| AC19I8_008420 | GT8 | GT |
| AC19I8_008451 | GH3 | GH |
| AC19I8_008498 | AA11 | AA |
| AC19I8_008519 | GH31 | GH |
| AC19I8_008557 | GT90 | GT |
| AC19I8_008558 | AA9 | AA |
| AC19I8_008640 | GT15 | GT |
| AC19I8_008652 | GT66 | GT |
| AC19I8_008796 | GH1 | GH |
| AC19I8_008834 | GH16 | GH |
| AC19I8_008864 | AA9 | AA |
| AC19I8_008878 | PL1 | PL |
| AC19I8_008880 | GH3 | GH |
| AC19I8_008883 | GH18 | GH |
| AC19I8_008886 | AA1 | AA |
| AC19I8_008896 | GH79 | GH |
| AC19I8_008921 | AA9 | AA |
| AC19I8_008932 | AA7 | AA |
| AC19I8_009031 | GT22 | GT |
| AC19I8_009073 | GH132 | GH |
| AC19I8_009124 | AA3 | AA |
| AC19I8_009150 | AA9 | AA |
| AC19I8_009211 | GH131 | GH |
| AC19I8_009220 | GH65 | GH |
| AC19I8_009271 | AA3 | AA |
| AC19I8_009287 | PL4 | PL |
| AC19I8_009290 | GH55 | GH |
| AC19I8_009315 | AA11 | AA |
| AC19I8_009330 | GT22 | GT |
| AC19I8_009334 | GT3 | GT |
| AC19I8_009344 | GT15 | GT |
| AC19I8_009375 | GH31 | GH |
| AC19I8_009398 | GH1 | GH |
| AC19I8_009410 | GH31 | GH |
| AC19I8_009451 | AA7 | AA |
| AC19I8_009460 | GT21 | GT |
| AC19I8_009484 | AA1 | AA |
| AC19I8_009505 | GH3 | GH |
| AC19I8_009512 | GH5 | GH |
| AC19I8_009620 | GH27 | GH |
| AC19I8_009651 | GH2 | GH |
| AC19I8_009652 | GH2 | GH |
| AC19I8_009703 | GH15 | GH |
| AC19I8_009742 | GT58 | GT |
| AC19I8_009742 | GT58 | GT |
| AC19I8_009763 | GT15 | GT |
| AC19I8_009782 | GH72 | GH |
| AC19I8_009810 | GH35 | GH |
| AC19I8_009826 | GH17 | GH |
| AC19I8_009907 | GH88 | GH |
| AC19I8_009931 | GT2_Glyco_tranf_2 | GT |
| AC19I8_009943 | CE1 | CE |
| AC19I8_009943 | CE1 | CE |
| AC19I8_009975 | GH17 | GH |
| AC19I8_010018 | GT2_Glycos_transf | GT |
| AC19I8_010018 | GT2_Glycos_transf | GT |
| AC19I8_010063 | GH43 | GH |
| AC19I8_010144 | AA3 | AA |
| AC19I8_010154 | GH17 | GH |
| AC19I8_010177 | AA3 | AA |
| AC19I8_010217 | AA7 | AA |
| AC19I8_010218 | AA9 | AA |
| AC19I8_010248 | GT59 | GT |
| AC19I8_010304 | GH53 | GH |
| AC19I8_010435 | GH43 | GH |
| AC19I8_010435 | GH43 | GH |
| AC19I8_010454 | GH115 | GH |
| AC19I8_010467 | GH31 | GH |
| AC19I8_010468 | GH13 | GH |
| AC19I8_010696 | AA7 | AA |
| AC19I8_010703 | AA7 | AA |
| AC19I8_010716 | CE4 | CE |
| AC19I8_010753 | AA12 | AA |
| AC19I8_011006 | GH32 | GH |
| AC19I8_011012 | CE12 | CE |
| AC19I8_011030 | AA7 | AA |
| AC19I8_011045 | CE5 | CE |
| AC19I8_011096 | GT34 | GT |
| AC19I8_011135 | CBM21 | CB |
| AC19I8_011217 | GH28 | GH |
| AC19I8_011237 | CE4 | CE |
| AC19I8_011242 | GH43 | GH |
| AC19I8_011551 | GH7 | GH |
| AC19I8_011594 | GH5 | GH |
